# Different Prostatic Tissue Microbiomes between High- and Low-Grade Prostate Cancer Pathogenesis

**DOI:** 10.3390/ijms25168943

**Published:** 2024-08-16

**Authors:** Jae Heon Kim, Hoonhee Seo, Sukyung Kim, Md Abdur Rahim, Sujin Jo, Indrajeet Barman, Hanieh Tajdozian, Faezeh Sarafraz, Ho-Yeon Song, Yun Seob Song

**Affiliations:** 1 Department of Urology, Soonchunhyang University School of Medicine, Seoul 04401, Republic of Korea; 2 Department of Microbiology and Immunology, School of Medicine, Soonchunhyang University, Asan 31151, Republic of Korea; 3 Human Microbiome Medical Research Center (HM-MRC), Soonchunhyang University, Asan 31538, Republic of Korea

**Keywords:** prostate cancer, high-grade prostate tumor (HGT), low-grade prostate tumor (LGT), biomarker, *Cutibacterium*, therapeutic targets

## Abstract

Numerous human pathologies, such as neoplasia, are related to particular bacteria and changes in microbiome constituents. To investigate the association between an imbalance of bacteria and prostate carcinoma, the microbiome and gene functionality from tissues of patients with high-grade prostate tumor (HGT) and low-grade prostate tumor (LGT) were compared utilizing next-generation sequencing (NGS) technology. The results showed abnormalities in the bacterial profiles between the HGT and LGT specimens, indicating alterations in the make-up of bacterial populations and gene functionalities. The HGT specimens showed higher frequencies of *Cutibacterium*, *Pelomonas*, and *Corynebacterium* genera than the LGT specimens. Cell proliferation and cytokine assays also showed a significant proliferation of prostate cancer cells and elevated cytokine levels in the cells treated with *Cutibacterium*, respectively, supporting earlier findings. In summary, the HGT and LGT specimens showed differences in bacterial populations, suggesting that different bacterial populations might characterize high-grade and low-grade prostate malignancies.

## 1. Introduction

In humans, more than 100 trillion microbial cells have a symbiotic relationship with their human host [1]. It is well-recognized that bacteria in specific body regions contribute to the integrity of the immune system, pathogenesis, and ongoing well-being. The innovation of high-throughput next-generation sequencing (NGS) methods has led to a rise in the number of studies being performed regarding the role played by the human microbiome in a range of pathologies [1].

In males, prostate carcinoma is one of the foremost neoplasias [2]. Potential contributing factors to prostate biology include infections by viruses and bacteria, inflammatory triggers such as diet and lifestyle, and environmental influences [3,4,5,6]. The well-being of the host is substantially influenced by the resident bacterial community, as the interplay between these microbial communities and their hosts indicates their participation in various biochemical activities which can influence carcinogenesis [7,8,9,10].

Concerning the development of malignant tissue within the prostate, potential influencing factors can either trigger or sustain inflammation within the tissue. Bacterial communities may cause or exacerbate the inflammatory process within carcinomatous prostate tissues [11]. Studies have shown that changes in the constituents of bacterial communities play a significant role in carcinogenesis by enhancing proinflammatory processes or altering the media outside prostatic cells [12,13,14].

Disease-inducing microorganisms deemed to promote the inflammatory process within the prostate can incorporate opportunistic endogenous Enterobacteriaceae (e.g., *Escherichia coli* or *Pseudomonas* spp.) and bacteria transmitted via sexual activity (i.e., *Neisseria gonorrhoeae*, *Chlamydia trachomatis*, and *Trichomonas vaginalis*) [15,16]. Augmented visceral inflammation has especially been recognized in specimens of prostate malignancy in the presence of *Propionibacterium acnes* (*Cutibacterium acnes*) [13,16,17]. Moreover, it is believed that microbial dysbiosis (alterations in the healthy microbiota) could play a crucial role in prostate cancer pathogenesis, although this area is still understudied [18]. In particular, altered microbiota can influence the normal immune system, thus preventing the effective surveillance of tumor growth [19]. Metabolic changes such as generating reactive oxygen species (ROS) due to dysbiosis can promote cancerous mutations [19,20]. Moreover, the altered microbiota can form biofilms that protect cancer cells from the immune system and therapeutic agents, thus enhancing cancer pathogenesis [21].

Several previous articles have detailed the microbiome in the male genital tract by performing prokaryotic and viral DNA sequence analyses from neoplastic prostate samples. Epidemiological studies showed that 87% of prostate cancer patients have microbial DNA in their prostates [22]. Studies reported the abundance of *Escherichia*, *Propionibacterium*, *Acinetobacter*, and *Pseudomonas* in the prostate microenvironment, thus constituting the core of the prostate microbiome [22]. Specifically, *P. acnes*, JC polyomavirus (JCV), and BK polyomavirus (BKV) have been more commonly detected in prostate cancer patients than in the controls [23,24,25], indicating the association of particular bacterial species in prostate cancer development. However, finding the causal relationships between the human microbiome and prostate cancer requires more comprehensive, longitudinal, and interventional studies. The interplay between the human microbiome and prostate cancer is highly complex. Understanding these interactions can provide insights into the pathways through which the microbiota influences cancer development, progression, and treatment responses. There have been many studies on prostate cancer and the microbiome, but few have looked at the association with the progression of the disease. Most of the research focuses only on the presence of the microbiome in the diet, not on cross-sectional cancers [26]. Though some studies classified the human microbiome within diseased and normal prostate samples [18,22,27], more in-depth and comprehensive studies are required to understand better the human microbiome’s role in prostate cancer development. This need is emphasized by the recent advances in high-throughput sequencing and bioinformatic technologies [28,29].

Many human pathologies, such as malignancy, are linked to specific bacteria and changes in microbiome constituents. To examine the association between an imbalance of bacteria and prostate carcinoma and its pertinence to the disease pathways underlying the tumor, microbiomes from patients with high-grade prostate tumors (HGTs) and low-grade prostate tumors (LGTs) were compared using NGS. 

## 2. Results

### 2.1. Construction of Metagenomic Library

**Figure 1:**
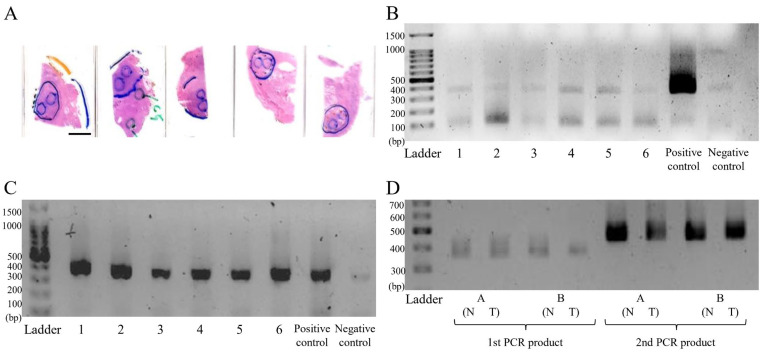
Metagenomic library construction for next-generation sequencing. (**A**) DNA was extracted from the formalin-fixed paraffin-embedded (FFPE) prostate tissue specimens. (**B**) Following this, amplicon PCR was conducted, showing poor visibility of the expected band. (**C**) Consequently, the PCR was repeated, showing the expected PCR size of 359 bp, similar to amplified DNA from the mice stool sample used as a positive control. (**D**) Subsequently, index PCR was conducted, showing the expected size of 457 bp (right panel of the image). Throughout the experiments, in every step from DNA extraction to metagenomic library construction and sequencing, all the DNA samples were quantified, adjusted, and qualified in contamination-free conditions to ensure the quality control of our experimental procedures. The scale bar on the FFPE images represents 3 cm.

The metagenomic library was constructed for sequencing. Figure 1A shows the formalin-fixed paraffin-embedded (FFPE) prostate tissue specimens. The initial amplicon PCR results showed the presence of bacterial DNA with poor visibility, whereas the repeated PCR results detected the expected PCR size of 359bp (Figure 1B,C). Figure 1D shows the agarose gel results of the first PCR product (amplicon PCR) and second PCR product (Index PCR) with expected sizes of 359 bp and 457 bp, respectively. These findings ensure the quality control or validation measures implemented during the experimental procedure.

### 2.2. Overall Structure of Bacterial Communities across Samples

**Figure 2:**
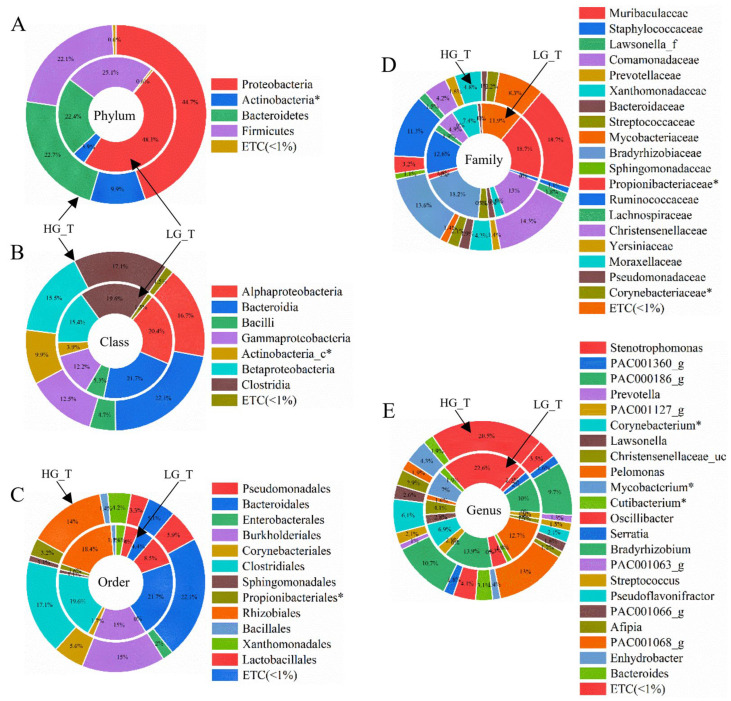
Averaged taxonomic composition for tumor regions in high-grade tumor (HGT, 2–4) or low-grade tumor (LGT, 0–1) groups. The taxonomic relative abundance in the HGT and LGT groups was further classified at levels of (**A**) phylum, (**B**) class, (**C**) order, (**D**) family, and (**E**) genus. The data showed a significant difference in the taxonomic relative abundance between the HGT and LGT groups at the following levels: phylum, Actinobacteria; class, Actinobacteria_c; order, Propionibacteriales; family, Corynebacteriaceae and Propionibacteriaceae; and genus, *Corynebacterium*, *Mycobacterium*, and *Cutibacterium*. The Wilcoxon rank-sum test was used to analyze the significance of the difference between the groups (*, *p* < 0.05). A relative abundance of less than 1% was expressed as ETC.

A total of 26 samples were sequenced using an Illumina iSeq100 system. A total of 1,336,478 reads were obtained after using a pre-filter to remove low-quality reads from the raw data generated by the NGS platform. After the non-specific amplicons, amplicons were not assigned to the target taxa. Chimeras were removed from the QC process. The total valid reads used for data analysis were 422,258. There was an average of 11,729 reads per sample (ranging from 441 to 41,829 reads), with an average length of 391 bp. After alignment, unique representative sequences were classified into 712 operation taxonomic units (OTUs) per sample at a 97% similarity level, from which 21 phyla, 43 classes, 95 orders, 207 families, and 806 genera were detected. Good’s coverage estimator was 97.34%, indicating that 16s rRNA sequences identified in this study likely represented the majority of bacterial sequences in the samples (Figure 2).

### 2.3. Bacterial Taxa (HGT vs. LGT)

The bacterial communities in HGT and LGT were analyzed at different taxonomic levels (Table S2, Figure 2A–E). A total of 21 phyla were identified, considering all OTUs defined. Proteobacteria, Bacteroidetes, and Firmicutes were the top three most abundant phyla. Together, their sequences accounted for 91.6% of all sequences in HGT. Proteobacteria, Firmicutes, and Bacteroidetes were the top three most abundant phyla in LGT. Together, their sequences accounted for 96.4% of all sequences. Proteobacteria comprised the most abundant phylum, accounting for 41.4% of HGT sequences and 41.7% of LGT sequences. Actinobacteria were more abundant in HGT than in LGT (*p* = 0.024).

A total of 43 classes were identified, considering all the OTUs defined. Bacteroidia, Betaproteobacteria, Gammaproteobacteria, and Clostridia were the top four most abundant classes of HGT. Together, their sequences accounted for 77.5% of all sequences in HGT. Bacteroidia, Alphaproteobacteria, Clostridia, Betaproteobacteria, and Gammaproteobacteria were the top five most abundant classes of LGT. Together, their sequences accounted for 88.6% of all sequences. Bacteroidia was the most abundant class, accounting for 30.9% of HGT and 31.8% of LGT sequences. Actinobacteria_*c* was more abundant in HGT than in LGT (*p* = 0.024).

A total of 95 orders were identified, considering all the OTUs defined. Bacteroidales, Burkholderiales, and Clostridiales were the top three most abundant orders. Together, their sequences accounted for 60.7% of all sequences in HGT. Bacteroidales, Burkholderiales, Clostridiales, and Pseudomodales were the top four most abundant orders of LGT. Together, their sequences accounted for 75.5% of all sequences. Bacteroidales was the most abundant order of HGT, accounting for 30.9% of HGT and 31.8% of LGT sequences. Propionibacteriales was more abundant in HGT than in LGT (*p* = 0.021).

A total of 207 families were identified, considering all the OTUs defined. Muribaculaceae and Comamonadacea were the top two most abundant orders. Together, their sequences accounted for 43% of all sequences in HGT. Muribaculaceae, Comamonadaceae, Ruminococcaceae, and Moraxellaceae were the top four most abundant orders of LGT. Together, their sequences accounted for 66.5% of all sequences. Muribaculaceae was the most abundant family in HGT and LGT, accounting for 26.3% of HGT and 27.5% of LGT sequences. Propionibacteriaceae (*p* = 0.031) and Corynebacteriaceae (*p* = 0.031) were more abundant in HGT than in LGT.

A total of 806 genera were identified, considering all the OTUs defined. *Pelomonas* and *PAC000186_g* were the top two most abundant genera. Together, their sequences accounted for 29.3% of all sequences in HGT. *Pelomonas* and *PAC000186_g* were the top two most abundant genera of LGT. Together, their sequences accounted for 30.6% of all sequences. *Pelomonas* was the most abundant genus in the HGT (15.6%) and LGT (15.6%) sequences. *Corynebacterium* (*p* = 0.031), *Mycobacterium*, and *Cutibacterium* (*p* = 0.031) were more abundant in HGT than in LGT.

### 2.4. Richness and Diversity (HGT vs. LGT)

**Figure 3:**
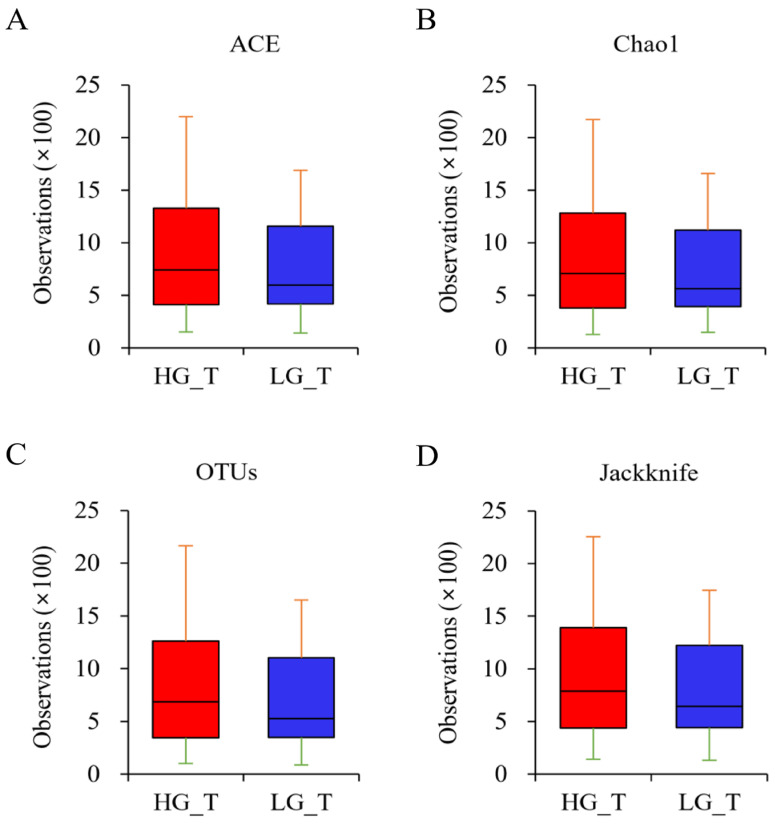
Boxplot of species richness indices. The species richness in the specimens from the HGT and LGT groups was analyzed with (**A**) Ace, (**B**) Chao1, (**C**) OTUs, and (**D**) Jackknife metrics. The data showed that the richness of the bacterial community was increased in the HGT samples compared to the LGT samples, although the difference was not statistically significant. The horizontal thick black band represents the median value. The margins of the boxplot indicate the first and third quartiles.

The richness of the bacterial community was explored utilizing Ace, Chao1, OTUs, and Jackknife metrics to estimate the α-diversity of the bacterial community (Figure 3A–D). The results showed that the richness of the bacterial community was increased in the HGT samples compared to the LGT samples, although the difference was not statistically significant.

**Figure 4:**
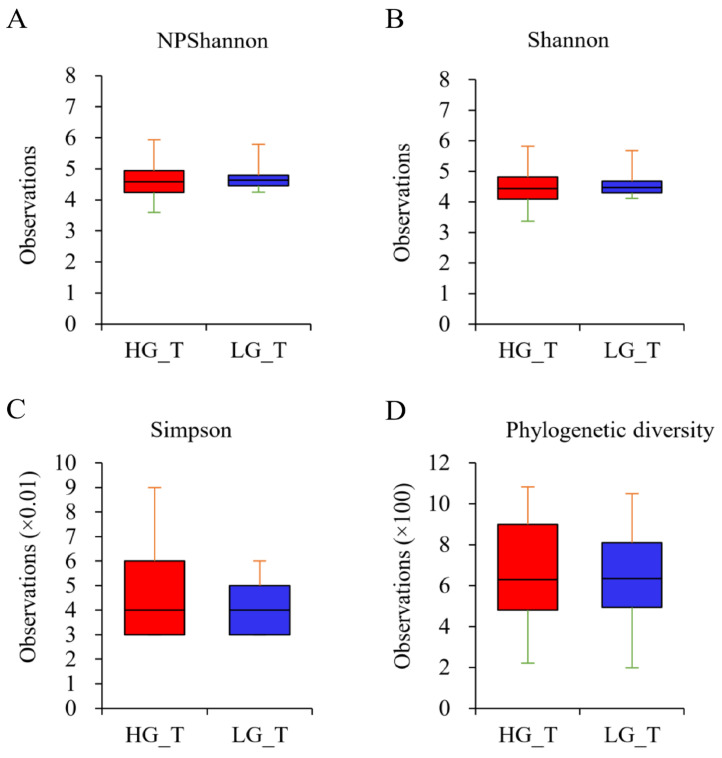
Boxplot of species diversity indices. The species diversity was investigated for samples from the HGT and LGT groups using the (**A**) NPShannon, (**B**) Shannon, (**C**) Simpson, and (**D**) Phylogenetic diversity metrics. The diversity of the bacterial community in the LGT samples was increased compared to that in the HGT samples. However, the difference was not statistically significant. The horizontal thick black band represents the median value. The margins of the boxplot indicate the first and third quartiles.

Furthermore, the α-diversity of the bacterial community was also investigated by analyzing the diversity of species present in specimens employing the NPShannon, Shannon, Simpson, and Phylogenetic diversity metrics (Figure 4A–D). The data revealed increased species diversity in the LGT samples compared to the HGT samples. However, the difference between the two was not statistically significant.

**Figure 5:**
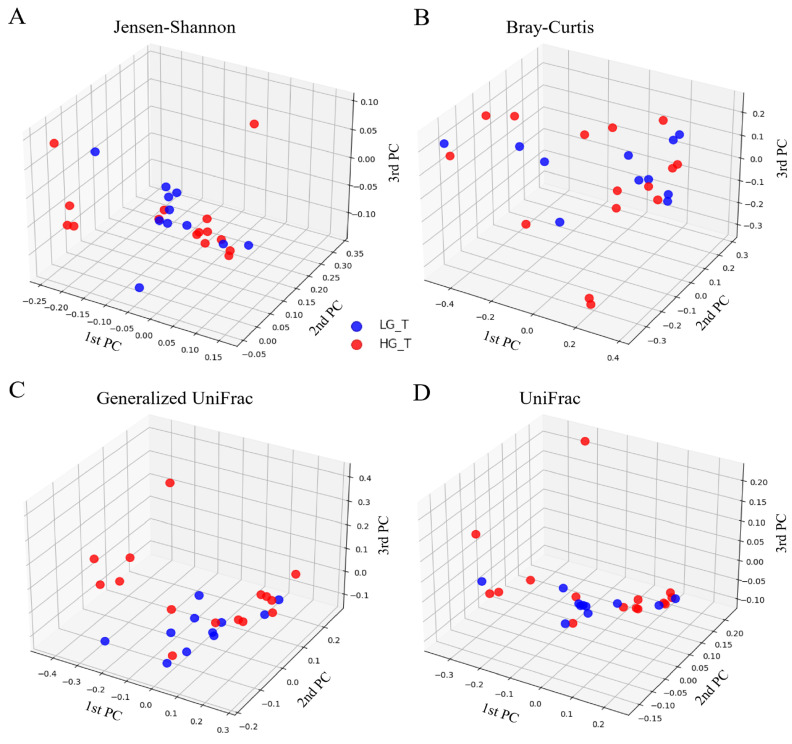
Principal coordinate analysis (PCoA) of the bacterial communities present in the low-grade tumor group (LGT) and high-grade tumor group (HGT). The extent of the diversity of the bacterial communities was analyzed using (**A**) Jansen–Shannon, (**B**) Bray–Curtis, (**C**) generalized UniFrac, and (**D**) UniFrac metrics at the OTU level. The data showed no apparent differences in the bacterial communities between the two groups.

The extent of the diversity of the bacterial communities was evaluated using Jensen–Shannon, Bray–Curtis, generalized UniFrac, and UniFrac principal component analysis (PCA) at the OTU level (Figure 5A–D). The results demonstrated no remarkable difference in the diversity of the bacterial communities between the two groups.

**Figure 6:**
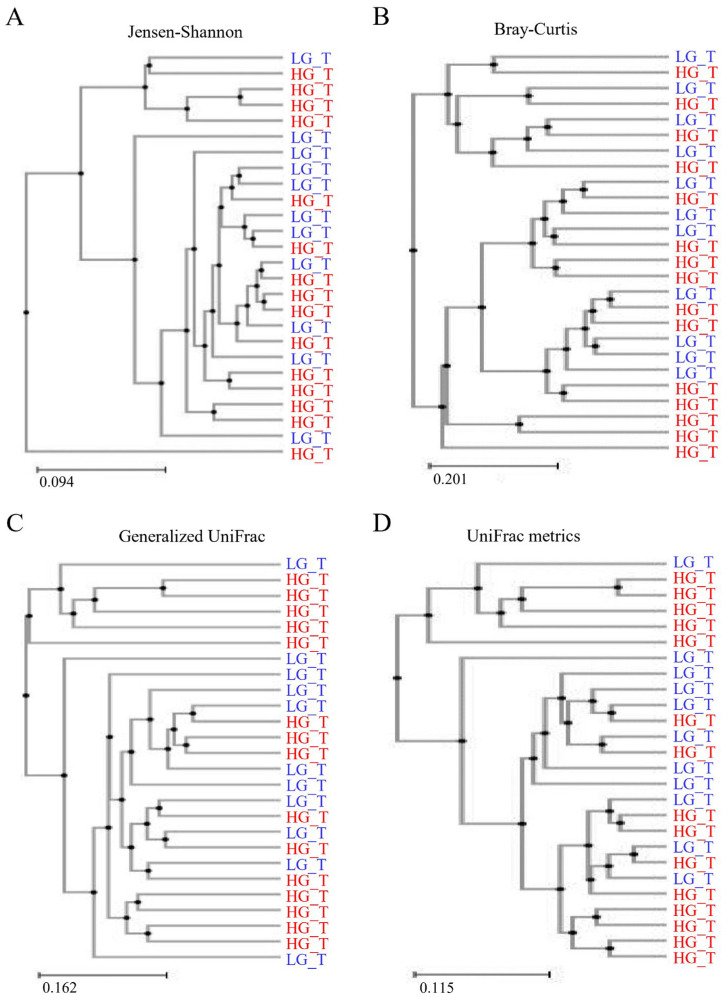
Clustering utilizing the Unweighted Pair Group Method with Arithmetic mean (UPGMA). The low-grade tumor (LGT) and high-grade tumor (HGT) groups were analyzed using (**A**) Jansen–Shannon, (**B**) Bray–Curtis, (**C**) Generalized UniFrac, and (**D**) UniFrac metrics. The findings suggest no significant structural difference in the bacterial communities between the HGT and LGT groups, as they did not cluster separately.

Clustering using the Unweighted Pair Group Method with Arithmetic mean (UPGMA) demonstrated that the bacterial communities in the HGT samples and LGT samples did not cluster separately, suggesting that the overall structures of the bacterial communities in these two groups were not different (Figure 6A–D). Spots representing the HGT samples presented more dispersed distribution patterns than those representing the LGT samples, consistent with the increased level of bacterial diversity found in the cancer samples.

Beta set-significance was demonstrated by a permutational multivariate analysis of variance (PERMANOVA) (Table 1). The beta-diversity analysis used the metrics of Jensen–Shannon, Bray–Curtis, Generalized UniFrac, and UniFrac. The results demonstrated no significant difference between the two groups.

### 2.5. LEfSe (HGT vs. LGT)

A linear discriminant analysis coupled with LEfSe was performed to further analyze the microbiota patterns of the HGT and LGT groups. The forest plot was generated from the LEfSe analysis, which showed the most differentially abundant taxa enriched in the microbiota (green for the LGT group and red for the HGT group).

**Figure 7:**
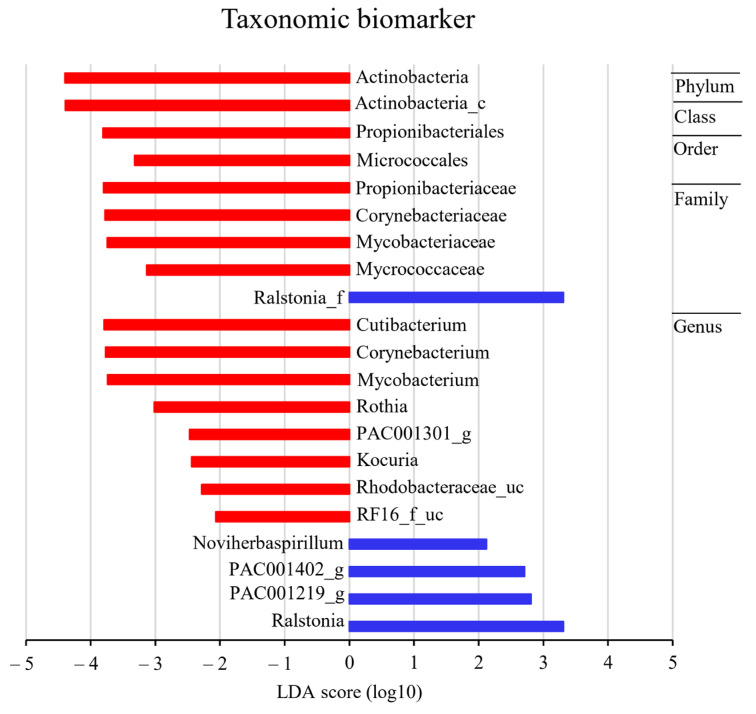
Discovery of taxonomic biomarkers for tumor (T) regions in the high-grade tumor (HGT, 2–4) or low-grade tumor (LGT, 0–1) groups using linear discriminant analysis effect size (LEfSe). The microbiota pattern between the HGT and LGT groups was analyzed using a linear discriminant analysis coupled with LEfSe. The results show the most differentially abundant taxa enriched in the microbiota, in blue for the LGT group and in red for the HGT group.

The bacterial compositions of the HGT samples varied from those of the LGT samples (Figure 7, Table S3). At the phylum level, Actinobacteria were significantly enriched in the HGT samples (LDA score ≤ −3). At the class level, Actinobacteria_c were significantly enriched in the HGT samples (LDA score ≤ −3). At the order level, Micrococcales and Propionibacteriales were significantly enriched in the HGT samples (LDA score ≤ −3). At the family level, Micrococcaceae, Mycobacteriaceae, Corynebacteriaceae, and Propionibactericeae were significantly enriched in the HGT samples (LDA score ≤ −3), while Ralstonia_f was significantly enriched in the LGT samples (LDA score ≥ 3). At the genus level, *Mycobacterium*, *Rothia*, *Corynebacterium*, and *Cutibacterium* were significantly enriched in the HGT samples (LDA score ≤ −3), while *Ralstonia* was significantly enriched in the LGT samples (LDA score ≥ 3).

### 2.6. PICRUSt (HGT vs. LGT)

**Figure 8:**
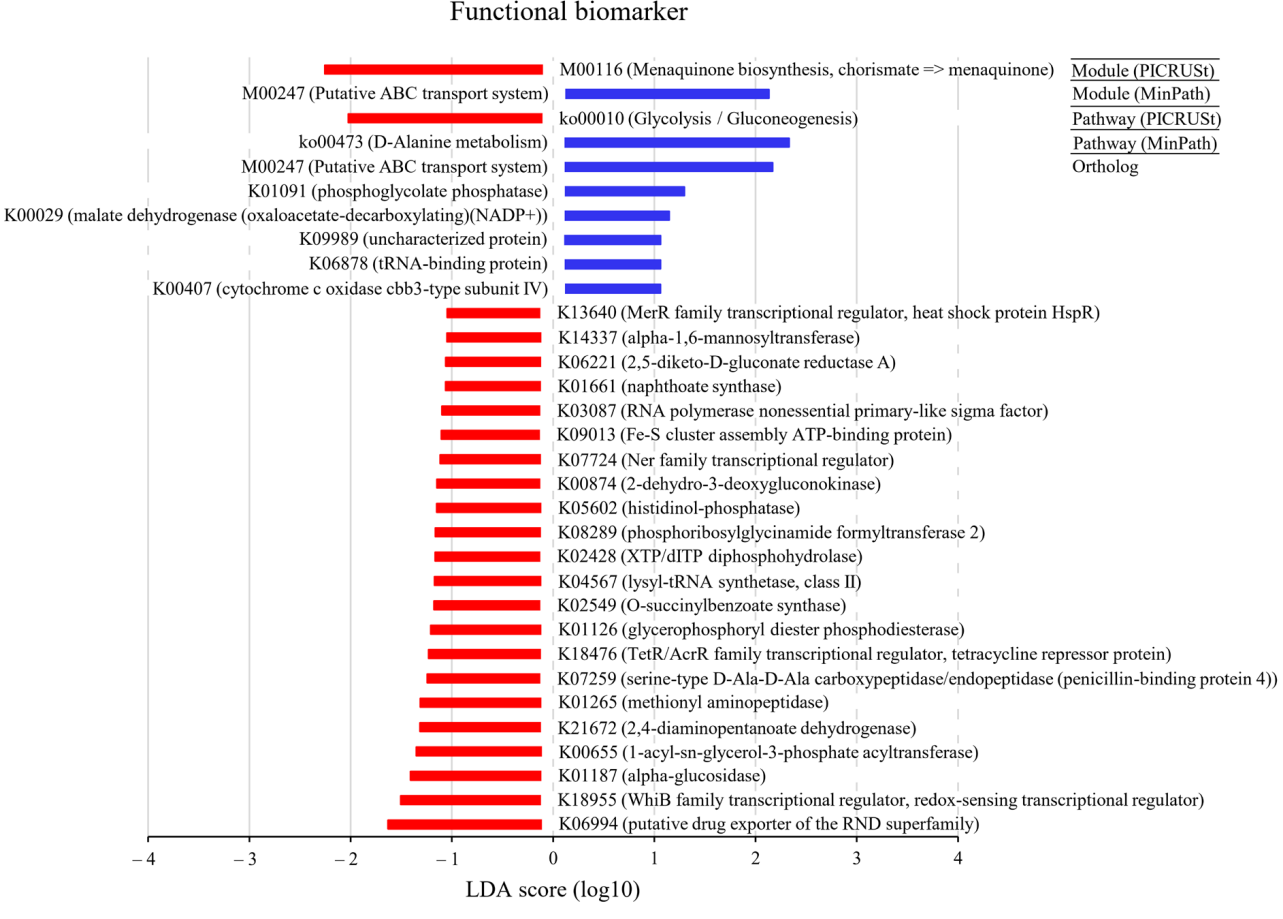
Discovery of functional biomarkers for tumor regions in the high-grade tumor (HGT, 2–4) and low-grade tumor (LGT, 0–1) groups using linear discriminant analysis effect size (LEfSe). Functional biomarkers were analyzed using ortholog, module, and pathway profiles. For module and pathway, the PICRUSt (Phylogenetic Investigation of Communities by Reconstruction of Unobserved States) and MinPath (Minimal set of Pathways) methods were applied, respectively. The KEGG (Kyoto Encyclopedia of Genes and Genomes) Database was used for the functional biomarker analysis. The data showed more abundance of the super pathway of glycolysis/gluconeogenesis in HGT than in LGT (LDA score ≤ −2). The red area indicates more abundance in the HGT group, and the blue area indicates more abundance in the LGT group.

The LEfSe outputs showed a series of metabolic pathways presenting significantly different distributions in each group (Figure 8, Table S4). Pathways related to genetic information processing were remarkably enriched in cancer lesions. PICRUSt was performed to explore the functional profiles of microbiota associated with prostate cancer. The super pathway of glycolysis/gluconeogenesis was more abundant in HGT than in LGT (LDA score ≤ −2).

### 2.7. Assessing the Impact of Cutibacterium on Cell Proliferation and Cytokine Production

**Figure 9:**
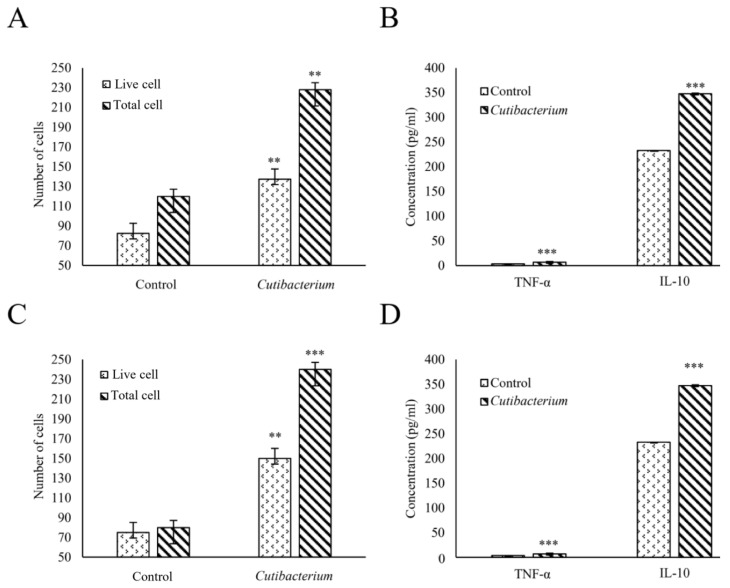
Evaluating the impact of *Cutibacterium* on prostate cancer cells. The effect of *Cutibacterium* on prostate cancer cells was explored using DU-145 and PC-3 cells. *Cutibacterium*-treated (**A**) DU-145 cells and (**C**) PC-3 cells were counted using a hemocytometer, showing significantly more proliferation than the untreated cells. Concurrently, the cytokine levels were also measured, revealing significant increases in TNF-α and IL-10 in the treated (**B**) DU-145 and (**D**) PC-3 cells compared to the untreated cells. The experiments were conducted in triplicate. The results are expressed as the mean ± standard deviation. The significance between groups was determined using an unpaired Student *t*-test (**, *p* < 0.01; ***, *p* < 0.001).

Cell proliferation and cytokine assays were conducted to explore the effect of *Cutibacterium* on prostate cancer cells (Figure 9). The cell proliferation results showed that the number of live DU-145 cells was significantly increased when they were treated with *Cutibacterium* compared to the untreated cells (*p* < 0.01) (Figure 9A). A significant increase in the total cell count was also observed in treated conditions compared to untreated conditions (*p* < 0.01). The cytokine assay revealed a significant increase in TNF-α in the *Cutibacterium*-treated group compared to the untreated group (*p* < 0.001) (Figure 9B). A significant increase in IL-10 was also observed in the treated DU-145 cells compared to the untreated cells (*p* < 0.001). A similar pattern of results was also observed when another prostate cancer cell, PC-3, was subjected to *Cutibacterium* treatment, and the number of live PC-3 cells increased significantly compared to the untreated cells (Figure 9C). Additionally, increased cytokine production was observed while treating PC-3 with *Cutibacterium* (Figure 9D). These findings elaborated on the potential mechanisms underlying the observed effects, including the possible involvement of inflammatory pathways and the modulation of the local microenvironment.

## 3. Discussion

The World Health Organisation’s categorization of *Helicobacter pylori* as a carcinogen has led to increased attention to the potential relationship between microorganisms and various phases of carcinogenesis [30]. Microbes have been suggested to be involved in the development of 15.4% of tumors in humans [31]. However, few data describe the role that such organisms play in the progression of prostate tumors.

Details have been published on a regional microbiome unique to the prostate [32]. The prostate contains numerous bacteria, implying a potential pathophysiological relationship between prostatic microbiome constituents and cancerous lesions per se [33]. One study has failed to find any differences in microbiomes between high- and low-grade prostate malignancies. Additionally, no relationships between the bacterial populations and the degree of cancer advancement were noted [33]. The absence of any association was attributed to the small population in this study. It does not exclude the possibility that the grade of malignancy might be related to dissimilar bacterial constituents within the microbiome [33].

To explore the link between bacterial imbalance and prostate carcinoma, we compared the microbiome and gene functionality of tissue samples from patients with high-grade prostate tumors (HGTs) and low-grade prostate tumors (LGTs) using next-generation sequencing (NGS) technology. Notably, while conducting the experiments, each step was validated to confirm the integrity of our obtained data. The results of this work indicated that Proteobacteria comprised the most frequently seen phylum. There was a greater population of Actinobacteria in HGT (*p* < 0.05). Concerning classes, the Bacteroidia class was the most commonly observed. A higher prevalence of Actinobacteria_c was noted in HGT (*p* < 0.05). Bacteriodales was the order showing the highest abundance in HGT. Propionibaceteriales more frequently arose in HGT than LGT (*p* < 0.05). Within both the HGT and LGT specimens, the family observed most often was Muribaculaceae. A higher prevalence of Propionibacteriaceae and Corynebacteriaceae was detected in HGT (*p* < 0.05). *Pelomonas* was the most frequently observed genus in both HGT and LGT. *Corynebacterium*, *Mycobacterium*, and *Cutibacterium* were more evident in HGT specimens (*p* < 0.05). The existence of a regional microbiome unique to the prostate was confirmed in this study. The HGT and LGT specimens exhibited differing bacterial populations.

The anatomical situation of the prostate means that it is accessible to bacteria originating from both dermatological and intestinal communities. Thus, microbes within the prostate might have been derived from either of these populations. The large abundance of *Cutibacterium* spp., comprising *C. acnes*, is consistent with the established part played by this bacterium in proinflammatory processes. It verifies the described relationship between *C. acnes* and prostate neoplasia [12,34,35]. The results of this study showed that, at the order and family hierarchical strata, the HGT specimens had higher abundances of Propionibacteriales and Propionibacteriaceae, supporting the theory that a larger population of *Propionibacterium* spp. is commonly evident in HGT. Thus, compared to LGT, HGT might be linked to the existence of these microorganisms.

The increased prevalence of *Corynebacteriaceae* within prostate disorders, essentially evidenced by the *Corynebacterium* spp., is in alignment with the ability of these bacteria to produce a biofilm and stick to extracellular matrix constituents, e.g., fibronectin. This process is frequently associated with the likelihood of tissue invasion [36]. These microorganisms are additionally well-recognized etiological factors in infections of the urinary tract or urethra [37]. In the current work, the *Corynebacteriaceae* family and the *Corynebacterium* genus were more abundant in HGT than in LGT. Thus, the microbiome microenvironments in HGT and LGT were observed to be dissimilar. There might be a relationship between the presence of the *Corynebacterium* spp. and HGT, but not between this bacterium and LGT.

Within the taxonomic hierarchy, an incremental alteration in the richness of several bacterial cohorts was observed in HGT and LGT. Trends toward an increased richness in the bacterial populations and greater heterogeneity were observed in the HGT and LGT samples, respectively. However, these patterns failed to reach statistical significance.

The PCA revealed no clear-cut separation between cohorts at the OTU level, indicating no variation between the bacterial populations within the HGT and LGT specimens. Clustering (UPGMA) failed to occur individually, indicating that the general configurations of the bacterial populations were similar between the HGT and LGT samples. This was affirmed by the beta-diversity analysis. Thus, the heterogeneity of the microbiome was unrelated to the grade of malignant lesion in this study.

Compared to the LGT specimens, the HGT samples showed greater enrichment for the following taxonomic hierarchical strata: phyla, actinobacteria; class, actinobacteria_c; order, micrococcales and propionibacteriales; family, micrococcales, mycobacteriaceae, cortnebacteriaceae, and propionibactericeae; and genus, *micrococcales*, *mycobacterium, rothia*, *cortnebacterium*, *cubibacterium*, and *propionibactericeae* (LDA scores ≤ −3 or ≥3). Thus, the current work linked a different microbiome constitutions to the malignancy grade.

The PICRUSt analysis revealed that the glycolysis and gluconeogenesis pathways were more plentiful in HGT than in the LGT specimens (LDA score ≤ −2). Thus, this research demonstrated that the grade of cancerous lesion was linked to a specific functional microbiome profile.

This study saw an imbalance in the bacterial population within the HGT specimens, as evidenced by alterations in the bacterial constituents and gene functionality when referenced against the LGT specimens. Specifically, the most common genus observed in HGT was *Pelomonas*. The HGT specimens also showed significantly higher *Corynebacterium* and *Cutibacterium* population densities than the LGT samples.

Considering that the *Cutibacterium* had the highest LDA score related to high-grade cancer, we conducted additional experiments, including cell proliferation and cytotoxicity assays, using different prostate cancer cell lines, including DU-145 and PC-3. Both cell lines were used for this investigation, as these cell lines are considered classical cell lines for prostate cancer research [38]. A significant increase in cell proliferation was observed when the cells were treated with *Cutibacterium*. This finding was consistent with previous studies reporting a close association between *C. acne* and cell carcinoma, which elevated the proliferative potential of these cells [39]. Moreover, a significant production of cytokines TNF-α and IL-10 was noticed in the *Cutibacterium*-treated cells, further indicating a close association of this bacterium with tumor activities. This was supported by studies showing these cytokines’ crucial role in prostate cancer development [40,41]. Based on these expanded findings, we propose that *Cutibacterium* may play a significant role in modulating the proliferative capacity of prostate cancer cells and the possible involvement of inflammatory pathways through mechanisms that warrant further investigation. Our findings in the in vitro assay and clinical samples suggest that targeting microbial interactions in the tumor microenvironment could represent a link between *Cutibacterium* and HGT and help develop a novel therapeutic approach in prostate cancer treatment.

LEfSe, used for biomarker analysis in this study, is a powerful computational bioinformatic tool designed to detect and interpret the differences in microbial communities between different sample groups [42]. Its ability to combine statistical testing with effect size estimation makes it a valuable tool in many areas of biological research [42,43]. This method ensures that the identified biomarkers are abundant in a particular group and biologically relevant for an individual study, providing robust insights into the microbial pattern linked to a specific disease severity [44,45,46,47,48]. In this study, LEfSe analysis identified *Cutibacterium* as the most significant biomarker for cancer differentiation, corroborating previous research and suggesting that this bacterium may play a vital role in prostate cancer progression [39,49]. Previous studies show the association of this bacterium with the proinflammatory microenvironment or their interaction with host cellular pathways, thereby enhancing tumor differentiation [50,51]. A key point is that microbiome datasets obtained from NGS have technical and analytical limitations; thus, such mathematical models may not always fully capture all the interactions within biological systems [52]. Nonetheless, understanding the roles of these bacteria can aid in developing targeted microbial therapies or diagnostic markers for the management of prostate cancer.

Our study has limitations, such as a limited number of samples from few participants. Additionally, the samples were collected from a specific geographic location, limiting the generalizability of our findings. Therefore, extensive, in-depth, comprehensive studies with more participants from different geographic locations must confirm the current findings. Despite these limitations, our study findings could add valuable information for understanding the interplay between the microbiome and carcinomas.

To summarize, a distinct alteration in microbial composition was observed between specimens collected from patients with LGT and HGT, indicating varied microbiome involvement concerning tumor severity.

## 4. Materials and Methods

### 4.1. Subject Recruitment and Sample Collection

We enrolled 26 individuals diagnosed with a prostate malignancy from the Urology Department of Soonchunhyang University Hospital in this study. This includes the same patient subjects as a previous study conducted by this research team [53]. The median age of the participants was 72.5 years. No patient had significant underlying conditions such as diabetes mellitus, immunocompromization, or genetic pathologies. No therapy for prostate malignancy had been prescribed. No anti-microbial agents had been administered for at least 14 days prior to the specimens being obtained. The patients selected for this study underwent a radical prostatectomy. Paraffin tissues were obtained through surgery. According to the Gleason grade system [30], the patients were divided into two groups: a low-grade group defined as prostate cancer patients with Gleason grades 1~2 and a high-grade group defined as prostate cancer patients with Gleason grades 3~5. The clinical characteristics of the patients are shown in Table S1. This study was conducted in compliance with the guidelines outlined in the Declaration of Helsinki [54], and it was approved by the Ethics Committee of Soonchunhyang University Hospital, Korea (approval number: 2017-02-002).

### 4.2. DNA Extraction

Metagenomic DNAs were extracted from the collected specimens using a QIAamp DNA Mini Kit (Qiagen, Hilden, Germany), following the manufacturer’s instructions. A quantitative assessment of the extracted DNAs was conducted using a NanoDrop ND-1000 spectrophotometer (Thermo Fisher Scientific, Waltham, MA, USA). The quantity and quality of the extracted DNA were determined using a Qubit-4 fluorometer (Thermo Fisher Scientific, Waltham, MA, USA) and the agarose gel electrophoresis technique, respectively. The DNA samples were stored at −20 °C until further analysis. During the experimental procedures and all the steps from DNA extraction to metagenomic library construction and sequencing, the DNA samples were quantified, adjusted, and qualified.

Additionally, all the procedures were performed under sterile conditions and included template-free controls.

### 4.3. Preparation of 16S rRNA Gene Amplicon Libraries

DNA samples were used to prepare 16S rRNA gene amplicon libraries for sequencing with an Illumina iSeq100 platform, following previously published protocols [55]. The V4 hypervariable section of the 16S rRNA gene was amplified using the 515 F (5′-GTGCCAGCMGCCGCGGTAA-3′) and 926 R (5′-CCGTCAATTYYTTTRAGTTT-3′) primers [56,57]. PCR reactions were conducted using a 2× KAPA HiFi HotStart ReadyMix (Kapa Biosystems, Wilmington, MA, USA), followed by clean-up using AMPure XPbeads (Beckman Coulter, UK). Next, following the manufacturer’s protocol, a metagenomic library was prepared using a Nextera XT DNA Library Prep Kit (Illumina, San Diego, CA, USA). Finally, the prepared library was loaded onto an iSeq reagent cartridge (Illumina) and sequenced on an iSeq platform (Illumina). The sequences were deposited in the Sequence Read Archive (SRA) (BioProject ID: PRJNA927108, accessible at https://www.ncbi.nlm.nih.gov/sra/PRJNA927108, accessed on 24 January 2023).

### 4.4. Illumina Sequencing and Bioinformatic Analysis of Metagenomic Data

The fast length adjustment of short reads (FLASH) software (version 1.2.11) was employed to amalgamate pairs of reads from the initial DNA sections [58]. The quantitative insights into microbial ecology (QIIME) software (version 1.9.1) was used for sequence analysis [59]. The assignment of sequences to operational taxonomic units (OTUs) was carried out with a 97% likeness. For individual OTUs, the respective illustrative sequences were highlighted, and taxonomic data were allocated with the RDP classifier [60]. Illustrative sequences were assigned to the taxonomy pyramid regarding the Human Microbiome Database, i.e., phylum to species. A Bayesian strategy was used, with a 97% cut-off parameter.

A sampling-based OTU analysis was conducted to determine bacterial heterogeneity, displayed as a rarefaction curve. The richness and spectrum of the bacteria populations within the specimens were assessed using α indices (i.e., Chao 1, ACE, Simpson, Shannon, and Good’s coverage), gauged at a 3% distance [61,62].

The heterogeneity of the bacteria within samples was compared using Student’s t-test. Unweighted UniFrac distance metrics-based PCA was carried out [63]. The interplay between the varying bacterial populations within the specimens was evaluated using the R package (version 4.1.3). Constituents of the bacterial populations within the specimens were interrogated using PLS-DA, a nonparametric analysis of Adonis distance matrices, and ANOSIM. Differing taxa between the two specimen types throughout the taxonomic strata were identified with the linear discriminant analysis effect size (LEfSe) (http://huttenhower.sph.harvard.edu/galaxy/, accessed on 24 January 2023) software. This software could also facilitate data presentation through taxonomic bar charts and cladograms [61]. Network configurations within the specimen’s bacterial communities were labeled using the Ecological Network Analysis Pipeline. Cytoscape was used for their presentation [64]. Within the two groups, bacterial functions were forecast using the Phylogenetic Investigation of Communities by Reconstruction of Unobserved States (PICRUSt) algorithm [65]. The MeV package (version MeV 4.9.0) facilitated data clustering and display. Bacterial populations’ operational constituents underwent prediction using PICRUSt in alignment with the dataset in the Kyoto Encyclopedia of Genes and Genomes (KEGG) [65,66]. The guidance, available at https://github.com/picrust/picrust2/wiki (accessed on 9 August 2022), was utilized to establish operational inferences of the microbiome, employing PICRUSt2 and OTUs. An analysis of variance permitted the recognition of any dissimilarities among the pathways [67]. 

### 4.5. Evaluating the Impact of Cutibacterium on Prostate Cancer Cells

The effects of *Cutibacterium* on prostate cancer cells were explored using the DU-145 and PC-3 cell lines. For this purpose, the bacterium *C. acne* (KCTC 5012) was purchased from the Korean Collection for Type Cultures (KCTC, Jeongeup, Republic of Korea) and grown in brain heart infusion broth (Kisan Bio, Seoul, Republic of Korea) under anaerobic conditions for 72 h at 37 °C. The DU-145 (KCLB, 30081) and PC-3 (KCLB, 21435) cell lines were purchased from the Korean cell line bank (KCLB) and cultured in RPMI 1640 media (Thermo Fisher Scientific, USA) supplemented with 10% heat-inactivated fetal bovine serum (Gibco, Waltham, MA, USA) and 1% penicillin-streptomycin (HyClone, Logan, UT, USA). The cells were maintained in a humidified atmosphere with 5% CO_2_ at 37 °C.

A cell proliferation assay was conducted to observe the impact of *Cutibacterium* on the DU-145 and PC-3 cells. The cells were seeded into 4-well plates (5 × 10^5^/well) and incubated under the abovementioned conditions. After reaching confluency, they were treated with *Cutibacterium* at 1 × 10^7^ CFU/mL and incubated for 24 h. Afterward, the cells were washed, stained with trypan blue (Gibco, USA), and counted with a hemocytometer (Marienfeld, Lauda-Königshofen, Germany) using an optical microscope (AX10, Carl Zeiss, Jena, Germany). Furthermore, the cytokine levels after treating the cells with *Cutibacterium* were investigated. For this purpose, the cells were seeded, treated, and incubated in the same way and conditions described earlier. After incubation, the suspension of treated cells was collected, and the cytokine TNF-α and IL-10 levels were determined using a Mouse ELISA Kit (Thermo Fisher Scientific, USA), following the manufacturer’s instructions. In this assay, the control-group cells were treated following the same conditions as the experimental group, except for the fact that they were not treated with *Cutibacterium*. All experiments were performed in triplicate to ensure the reliability and reproducibility of the results.

## 5. Conclusions

When evaluated against the LGT specimens, the HGT samples showed bacterial population alterations and differences in microorganism gene functionality. Thus, high-grade and low-grade prostate malignancy lesions might be associated with varied bacterial populations. However, to ensure the validity of the current findings, more extensive, multicenter, longitudinal, and mechanistic studies are required to reveal the causal relationship between these microbiota changes and cancer progression. Moreover, it is essential to fully characterize the whole microbial community and provide functional insights to achieve a more comprehensive understanding regarding the association of microbiota and prostate cancer. Furthermore, a detailed understanding of the interactions between microbiome and host factors is crucial for uncovering the mechanisms by which microbiota influence tumor initiation, progression, and metastasis. 

## Figures and Tables

**Table 1:** Statistically significant analysis of beta-diversity.

Pair-Wise	Genus	Species
Jensen–Shannon	N.S. (*p* = 0.449)	N.S. (*p* = 0.366)
Bray–Curtis	N.S. (*p* = 0.769)	N.S. (*p* = 0.752)
Generalized UniFrac	N.S. (*p* = 0.203)	N.S. (*p* = 0.228)
UniFrac	N.S. (*p* = 0.360)	N.S. (*p* = 0.357)

The permutational multivariate analysis of variance (PERMANOVA) results demonstrated the beta-diversity set between the high-grade tumor (HGT, 2–4) and low-grade tumor (LGT, 0–1) groups. N.S., not significant.

## Data Availability

The data contained in this article and the original data supporting the present study’s findings are available from the corresponding author upon reasonable request.

## Supplementary Materials

The following supporting information can be downloaded at https://www.mdpi.com/article/10.3390/ijms25168943/s1.

## Abbreviations

High-grade tumors (HGTs); low-grade tumors (LGTs); next-generation sequencing (NGS); permutational multivariate analysis of variance (PERMANOVA); principal coordinates analysis (PCoA); unweighted pair group method with arithmetic mean (UPGMA); linear discriminant analysis effect size (LEfSe); phylogenetic investigation of communities by reconstruction of unobserved states (PICRUSt); minimal set of pathways (MinPath); and Kyoto Encyclopedia of Genes and Genomes (KEGG).
