# Supplementary material for: Different Prostatic Tissue Microbiomes between High- and Low-Grade Prostate Cancer Pathogenesis
Source: Int J Mol Sci. 2024 Aug 16;25(16):8943. doi: 10.3390/ijms25168943 (PMC11354394; doi:10.3390/ijms25168943)
Supplement: Supplementary file 1 [file ijms-25-08943-s001.zip › ijms-3104602-supplementary.pdf]

Supplementary data

Table S1. Clinical characteristics of patients analyzed in this study

	LG_T (n=11)	HG_T (n=15)	Patient (n=26)
Age	70.9±5.9	71.5±6.2	71.2±6.0
Survival period (day)	1572.6±1046.5	1466.7±1084.0	1511.5±1048.3
Gleason score	6.6±0.7	8.1±0.9	7.5±1.1
Grade group	0, 1 (0.7±0.5)	2, 3, 4 (3.1±0.9)	2.1±1.4
Risk stratification	1.0±0.8	1.7±0.5	1.4±0.7
Pathologic T stage	1.3±0.8	0.5±0.7	0.8±0.8

LGT: Tumor region of low-grade group, HGT: Tumor region of high-grade group.

Table S2. Averaged taxonomic composition for tumor regions in the high-grade tumor (HGT, 2-4) and low-grade tumor (LGT, 0-1) groups

Taxon rank	Taxon name	LGT							HGT						
		Ave	SD	Min	Max	Q1	Q2	Q3	Ave	SD	Min	Max	Q1	Q2	Q3
Phylum	Proteobacteria	41.7	12.4	25.5	65.5	35.8	39.1	43.4	41.4	20.0	18.9	79.5	22.6	34.9	64.5
	Actinobacteria*	3.1	1.7	1.5	6.1	1.9	2.3	4.5	8.1	7.1	1.8	23.0	2.7	4.3	15.6
	Bacteroidetes	32.3	12.3	8.8	52.1	29.7	32.8	40.9	31.4	21.5	1.8	61.2	3.5	34.7	51.9
	Firmicutes	22.4	3.6	15.4	27.5	20.6	22.8	24.3	18.8	6.1	8.8	27.8	14.1	20.9	23.7
Class	Alphaproteobacteria	7.7	5.2	2.9	20.0	3.4	6.4	9.3	7.0	5.0	2.7	22.1	4.2	5.8	8.2
	Bacteroidia	31.8	12.6	7.6	51.9	29.1	32.3	40.8	30.9	21.7	1.0	60.7	2.5	34.1	51.9
	Bacilli	7.1	5.7	1.6	18.2	1.8	4.6	9.9	6.2	3.6	1.1	12.3	3.6	5.8	8.8
	Actinobacteria_c*	3.1	1.7	1.5	6.1	1.9	2.3	4.5	8.1	7.1	1.8	23.0	2.7	4.3	15.6
	Gammaproteobacteria	15.5	5.8	7.6	24.5	10.6	14.3	21.8	16.3	11.4	4.1	50.3	8.0	13.4	20.9
	Betaproteobacteria	18.5	10.3	5.9	45.1	10.9	17.6	21.6	18.0	9.6	8.5	36.8	10.1	12.1	23.4
	Clostridia	15.1	5.1	7.6	22.6	9.2	15.6	19.0	12.3	7.9	1.0	21.5	1.8	14.8	18.9
Order	Pseudomonadales	10.6	4.3	2.3	17.0	7.9	11.7	13.4	7.4	5.1	1.8	15.5	3.0	5.2	12.3
	Bacteroidales	31.8	12.6	7.6	51.9	29.1	32.3	40.8	30.9	21.7	1.0	60.7	2.5	34.1	51.9
	Enterobacterales	1.2	0.6	0.4	2.5	0.6	1.1	1.6	3.9	5.5	0.3	20.2	0.9	2.0	3.0
	Burkholderiales	18.0	10.2	5.3	44.7	10.8	17.1	20.8	17.5	9.4	6.9	34.4	9.9	11.8	22.9
	Corynebacteriales	1.3	0.7	0.6	2.4	0.6	1.2	2.0	4.4	5.6	0.6	18.1	0.9	1.6	5.3
	Clostridiales	15.1	5.1	7.6	22.4	9.1	15.6	19.0	12.3	7.9	1.0	21.5	1.6	14.8	18.9
	Sphingomonadales	0.9	1.8	0.1	6.4	0.2	0.3	0.5	0.8	1.3	0.1	5.5	0.3	0.4	0.6
	Propionibacteriales*	1.4	0.9	0.5	3.2	0.7	1.0	1.8	2.8	2.3	0.8	9.4	1.5	2.2	2.9
	Rhizobiales	6.2	3.9	2.3	13.7	2.8	5.3	8.3	4.6	2.6	2.2	11.7	2.4	3.9	6.3
	Bacillales	2.1	1.7	0.6	6.7	0.8	1.7	2.4	2.2	1.9	0.2	6.9	0.7	2.0	2.3
	Xanthomonadales	3.2	2.9	0.6	9.7	0.7	2.2	4.3	4.4	6.1	0.3	24.5	1.0	2.3	6.4
	Lactobacillales	5.0	4.5	1.0	15.8	1.1	3.1	7.7	4.1	2.2	0.9	9.0	2.8	3.7	5.4
Family	Muribaculaceae	27.5	11.6	4.9	45.3	22.6	27.9	36.7	26.3	18.6	0.4	52.7	1.4	29.0	44.0
	Staphylococcaceae	1.7	1.5	0.5	5.7	0.6	1.3	1.8	1.8	1.7	0.2	6.2	0.6	1.5	1.8
	Lawsonella_f	0.5	0.3	0.0	1.2	0.2	0.4	0.7	1.2	3.4	0.1	13.4	0.1	0.3	0.7
	Comamonadaceae	15.9	10.6	4.6	44.2	8.9	14.9	18.9	16.7	9.3	6.4	33.9	9.3	11.2	22.6
	Christensenellaceae	4.0	1.8	0.9	6.7	2.2	4.7	5.0	3.2	2.4	0.1	6.5	0.3	3.6	5.1
	Xanthomonadaceae	3.2	2.9	0.6	9.7	0.7	2.2	4.3	4.4	6.1	0.3	24.5	1.0	2.3	6.4
	Bacteroidaceae	3.1	1.4	0.7	5.8	2.1	2.9	3.8	2.9	2.3	0.2	7.0	0.4	2.7	4.6
	Streptococcaceae	3.7	4.2	0.5	14.7	0.7	1.8	4.3	2.6	1.9	0.5	7.7	1.4	2.0	3.9
	Prevotellaceae	1.0	0.9	0.4	3.5	0.5	0.7	1.2	1.6	1.6	0.1	5.4	0.4	0.9	2.1
	Bradyrhizobiaceae	5.9	4.0	1.9	13.5	2.2	5.1	7.9	4.2	2.6	1.4	11.4	2.1	3.6	6.0
	Sphingomonadaceae	0.9	1.8	0.1	6.3	0.2	0.3	0.5	0.8	1.3	0.1	5.5	0.3	0.4	0.6
	Propionibacteriaceae*	1.4	0.9	0.5	3.2	0.7	1.0	1.8	2.8	2.3	0.8	9.4	1.5	2.2	2.9
	Mycobacteriaceae*	0.0	0.0	0.0	0.0	0.0	0.0	0.0	1.4	4.3	0.0	13.6	0.0	0.0	0.1
	Lachnospiraceae	2.8	0.9	1.5	4.2	1.8	3.0	3.5	2.3	1.3	0.1	3.7	1.0	2.7	3.3
	Pseudomonadaceae	1.6	1.6	0.2	6.0	0.6	1.4	1.6	1.7	2.9	0.2	12.0	0.4	0.9	1.4
	Yersiniaceae	1.0	0.6	0.4	2.4	0.5	1.0	1.4	3.6	5.4	0.3	19.7	0.6	1.7	2.9
	Corynebacteriaceae*	0.8	0.5	0.3	1.9	0.4	0.5	1.4	2.1	2.0	0.3	6.6	0.6	1.2	4.1
	Ruminococcaceae	8.2	2.7	3.7	12.8	5.6	8.6	10.2	6.9	4.3	0.7	12.3	1.4	8.3	10.2
	Moraxellaceae	9.0	4.5	1.6	15.5	6.9	9.1	11.8	5.7	4.9	1.4	15.1	1.7	3.3	10.7
Genus	Stenotrophomonas	2.9	3.0	0.4	9.5	0.5	2.0	4.3	3.9	6.2	0.2	24.4	0.8	2.1	5.3
	Oscillibacter	2.5	1.0	0.7	3.7	1.6	2.2	3.6	2.3	1.4	0.2	3.9	0.6	2.8	3.5
	PAC000186_g	15.0	5.8	3.1	22.9	12.0	15.8	18.7	13.7	9.8	0.2	28.8	1.1	16.8	21.1
	Prevotella	1.0	0.9	0.4	3.5	0.5	0.7	1.2	1.5	1.6	0.1	5.0	0.4	0.9	2.0
	Christensenellaceae_uc	1.2	0.5	0.3	2.2	0.9	1.2	1.4	1.0	0.6	0.0	2.0	0.5	1.0	1.4
	Corynebacterium*	0.8	0.5	0.3	1.8	0.4	0.5	1.4	2.1	1.9	0.3	6.4	0.6	1.2	4.1
	Lawsonella	0.5	0.3	0.0	1.2	0.2	0.4	0.7	1.2	3.4	0.1	13.4	0.1	0.3	0.7
	PAC001360_g	1.7	0.8	0.5	3.1	0.6	1.9	2.3	1.4	1.0	0.0	2.6	0.7	1.2	2.4
	Pelomonas	15.6	10.7	4.5	44.2	8.6	14.2	18.6	15.6	9.3	6.3	33.5	9.0	10.6	21.2
	Mycobacterium*	0.0	0.0	0.0	0.0	0.0	0.0	0.0	1.7	4.7	0.0	13.4	0.0	0.0	0.2
	Cutibacterium*	1.4	0.9	0.5	3.2	0.7	1.0	1.8	2.7	2.3	0.8	9.4	1.5	2.2	2.9
	PAC001127_g	2.8	1.3	0.3	4.8	2.3	2.8	3.7	2.4	1.3	0.0	3.6	1.4	3.2	3.2
	Serratia	1.0	0.6	0.4	2.4	0.5	0.9	1.4	3.6	5.4	0.2	19.6	0.6	1.6	2.9
	Bacteroides	3.1	1.4	0.7	5.8	2.1	2.9	3.8	2.9	2.3	0.2	7.0	0.4	2.7	4.6
	PAC001063_g	1.1	1.1	0.0	3.7	0.4	1.0	1.5	1.6	1.2	0.0	4.0	0.6	1.3	2.4
	Streptococcus	3.7	4.2	0.5	14.7	0.7	1.8	4.2	2.5	1.9	0.5	7.6	1.4	2.0	3.8
	Pseudoflavonifractor	4.2	1.9	1.4	8.4	2.9	4.5	4.9	3.4	2.3	0.1	6.9	0.7	4.1	5.2
	PAC001066_g	4.0	1.4	1.2	5.8	3.5	4.2	5.3	3.6	2.7	0.1	8.3	0.4	4.2	5.0
	Afipia	1.3	1.0	0.3	3.5	0.6	1.0	1.9	0.9	0.7	0.2	2.7	0.4	0.8	1.2
	PAC001068_g	2.2	3.2	0.1	11.6	0.6	1.4	1.9	2.7	3.4	0.0	10.5	0.2	1.4	3.6
	Enhydrobacter	8.4	4.6	1.0	14.7	5.3	8.7	11.8	4.9	4.9	0.8	14.8	1.3	2.2	9.7
	Bradyrhizobium	4.6	3.1	1.4	10.7	1.6	3.7	6.0	3.3	2.0	1.1	8.6	1.8	2.8	4.8

Relative abundances less than 1 % were expressed as ETC. Wilcoxon rank-sum test was used to analyze the significance between the two groups (*, $p < 0.05$). Ave, average; SD, standard deviation; Min, minimum; Max, maximum; Q1, first quartile; Q2: median; Q3, third quartile.

Table S3. Taxonomic biomarkers for tumor (T) regions in high-grade tumor (HGT, 2-4) and low-grade tumor (LGT, 0-1) groups

Taxon name	Taxon rank	LDA score (log10)	p-value	HGT	LGT
Actinobacteria	Phylum	-4.39547	0.02397	9.9029	3.8635
Actinobacteria_c	Class	-4.39299	0.02397	9.8648	3.8562
Propionibacteriales	Order	-3.80876	0.02091	3.1854	1.5983
Micrococcales	Order	-3.31964	0.04036	0.998	0.5536
Propionibacteriaceae	Family	-3.80156	0.03125	3.1515	1.5871
Corynebacteriaceae	Family	-3.77613	0.03125	2.1643	0.8376
Mycobacteriaceae	Family	-3.74387	0.03753	1.4205	0.0077
Micrococcaceae	Family	-3.13463	0.02091	0.5532	0.2635
Ralstonia_f	Family	3.30935	0.0457	0.3749	0.7467
Cutibacterium	Genus	-3.79158	0.03125	3.0923	1.5661
Corynebacterium	Genus	-3.76831	0.03125	2.1303	0.829
Mycobacterium	Genus	-3.73659	0.03753	1.3976	0.0077
Rothia	Genus	-3.01635	0.01818	0.4475	0.2275
PAC001301_g	Genus	-2.46963	0.03516	0.0834	0.0245
Kocuria	Genus	-2.43788	0.01504	0.0663	0.017
Rhodobacteraceae_uc	Genus	-2.28113	0.04895	0.0493	0.0094
RF16_f_uc	Genus	-2.06417	0.03807	0.0217	0
Noviherbaspirillum	Genus	2.11913	0.03926	0.0094	0.0343
PAC001402_g	Genus	2.70862	0.0182	0.2013	0.3077
PAC001219_g	Genus	2.81	0.03968	0.1634	0.3045
Ralstonia	Genus	3.31002	0.04036	0.3697	0.7432

Taxonomic biomarkers analysis for tumor (T) regions in the high-grade tumor (HGT, 2-4) or low-grade tumor (LGT, 0-1) groups using Linear discriminant analysis Effect size (LEfSe).

Table S4. Functional biomarkers for tumor (T) regions in high-grade tumor (HGT, 2-4) and low-grade tumor (LGT, 0-1) groups

Definition	Ortholog	LDA score (log10)	p-value	HGT	LGT
ko00473 (D-Alanine metabolism)	Pathway (MinPath)	2.33125	0.0457	0.5152	0.5528
ko00010 (Glycolysis / Gluconeogenesis)	Pathway (PICRUSt)	-2.02227	0.0102	0.6566	0.6352
M00247 (Putative ABC transport system)	Module (MinPath)	2.13206	0.01369	0.2132	0.2418
M00116 (Menaquinone biosynthesis, chorismate => menaquinone)	Module (PICRUSt)	-2.25466	0.00468	0.3831	0.3497
M00247 (Putative ABC transport system)	Ortholog	2.16803	0.0457	0.209	0.2385
K06994 (putative drug exporter of the RND superfamily)	Ortholog	-1.63037	0.0274	0.0127	0.0044
K18955 (WhiB family transcriptional regulator, redox-sensing transcriptional regulator)	Ortholog	-1.50463	0.0102	0.009	0.0032
K01187 (alpha-glucosidase)	Ortholog	-1.40773	0.00201	0.0263	0.0214
K00655 (1-acyl-sn-glycerol-3-phosphate acyltransferase)	Ortholog	-1.35108	0.00877	0.0479	0.0433
K21672 (2,4-diaminopentanoate dehydrogenase)	Ortholog	-1.31427	0.01581	0.0053	0.0012
K01265 (methionyl aminopeptidase)	Ortholog	-1.31181	0.01369	0.0556	0.0512
K07259 (serine-type D-Ala-D-Ala carboxypeptidase/endopeptidase (penicillin-binding protein 4))	Ortholog	-1.24286	0.02397	0.0131	0.01
K18476 (TetR/AcrR family transcriptional regulator, tetracycline repressor protein)	Ortholog	-1.22981	0.01581	0.0074	0.0041
K01126 (glycerophosphoryl diester phosphodiesterase)	Ortholog	-1.20888	0.02091	0.0787	0.075
K02549 (O-succinylbenzoate synthase)	Ortholog	-1.17616	0.00025	0.009	0.0061
K04567 (lysyl-tRNA synthetase, class II)	Ortholog	-1.17154	0.02397	0.0232	0.0201
K02428 (XTP/dITP diphosphohydrolase)	Ortholog	-1.16507	0.01183	0.0274	0.0248
K08289 (phosphoribosylglycinamide formyltransferase 2)	Ortholog	-1.16277	0.00468	0.0096	0.0071
K05602 (histidinol-phosphatase)	Ortholog	-1.14967	0.03125	0.0038	0.0016
K00874 (2-dehydro-3-deoxygluconokinase)	Ortholog	-1.14752	0.02091	0.0338	0.0307
K07724 (Ner family transcriptional regulator)	Ortholog	-1.11495	0.02397	0.0071	0.0046
K09013 (Fe-S cluster assembly ATP-binding protein)	Ortholog	-1.10376	0.00141	0.0068	0.0044
K03087 (RNA polymerase nonessential primary-like sigma factor)	Ortholog	-1.09755	0.03556	0.0083	0.006
K01661 (naphthoate synthase)	Ortholog	-1.0613	0.00031	0.0079	0.0057
K06221 (2,5-diketo-D-gluconate reductase A)	Ortholog	-1.06026	0.01581	0.0098	0.0076
K14337 (alpha-1,6-mannosyltransferase)	Ortholog	-1.04805	0.0102	0.0035	0.0015
K13640 (MerR family transcriptional regulator, heat shock protein HspR)	Ortholog	-1.04536	0.01183	0.0031	0.0013
K00407 (cytochrome c oxidase cbb3-type subunit IV)	Ortholog	1.06088	0.03556	0.0157	0.0183
K06878 (tRNA-binding protein)	Ortholog	1.06155	0.01183	0.0175	0.0195
K09989 (uncharacterized protein)	Ortholog	1.06202	0.03556	0.0252	0.0277
K00029 (malate dehydrogenase (oxaloacetate-decarboxylating)(NADP+))	Ortholog	1.14709	0.0457	0.0384	0.0408

K01091 (phosphoglycolate phosphatase)	Ortholog	1.29679	0.00201	0.1047	0.1089

Functional biomarkers analysis for tumor regions in the high-grade tumor (HGT, 2-4) and low-grade tumor (LGT, 0-1) groups using Linear discriminant analysis Effect size analysis (LEfSe). The KEGG (Kyoto Encyclopedia of Genes and Genomes) Database was used for functional biomarker analysis.
